# Extensive Heterotopic Ossification in a Large Incisional Ventral Hernia After a Burn Injury Requiring Transversus Abdominis Release

**DOI:** 10.7759/cureus.35312

**Published:** 2023-02-22

**Authors:** Camila Franco Mesa, Sergio Mazzola Poli de Figueiredo, Richard Lu

**Affiliations:** 1 Surgery, University of Texas Medical Branch, Galveston, USA

**Keywords:** incisional ventral hernia, incisional hernia, abdominal wall reconstruction, major burn, heterotopic ossification

## Abstract

Heterotopic ossification (HO) is an atypical complication of burn injuries presenting in 0.2-4% of cases. Usually, HO develops surrounding long bones or joints after orthopedic procedures or trauma. However, on extremely rare occasions, HO can develop from other bones such as the xiphoid. The purpose of this case report is to describe a case of an open retromuscular abdominal wall reconstruction with bilateral transversus abdominis release (TAR) in a patient with extensive abdominal heterotopic ossification following a midline laparotomy in the setting of a large burn injury.

The patient was a 42-year-old man with a history of 55% total burn surface area (TBSA) second- and third-degree flame burns who was treated in a large academic hospital with a renowned burn unit. His case in particular was brought to attention for the rare presentation of the aftermath of a burn injury and the technical surgical challenge it posed. Five months after the last surgical intervention, the patient is doing well without further complications or clinical signs of hernia recurrence.

Since there are no established guidelines for patients with HO after burn injuries, learning about alternate strategies will expand the armamentarium of abdominal wall reconstruction surgeons in this challenging patient population. Specifically, retromuscular ventral hernia repair with transversus abdominis release and synthetic mesh can be used in complex ventral hernia repair complicated by heterotopic ossification after a major burn.

## Introduction

Heterotopic ossification (HO) refers to the formation of ectopic bone in soft tissue. This condition has been described as an atypical complication of burn injuries presenting in 0.2-4% of cases [1,2]. Factors such as total burn surface area, number of surgical interventions, amount of graft used, and presence of sepsis have been associated with HO [2]. Trauma and orthopedic procedures are usually related to HO; however, in extremely rare cases, this can develop from structures such as the xiphoid [3]. In this situation, the calcified mass lodges in the abdominal wall and even intraperitoneal organs, acting as a foreign body causing discomfort, non-healing lesions, and increased risk of infection, and making future surgical interventions challenging [4,5]. Procedures such as abdominal wall hernia repair in these patients are complex due to the distorted anatomy related to the calcified foreign body in the layers of the abdominal wall [5]. The presence of HO in patients with ventral hernias is rarely described in the literature [5], thus knowledge gaps in the matter remain present. To help address this gap, we describe a case of open retromuscular abdominal wall reconstruction with bilateral transversus abdominis release (TAR) in a patient with extensive abdominal heterotopic ossification following a midline laparotomy in the setting of a large burn injury.

## Case presentation

**Figure 1:**
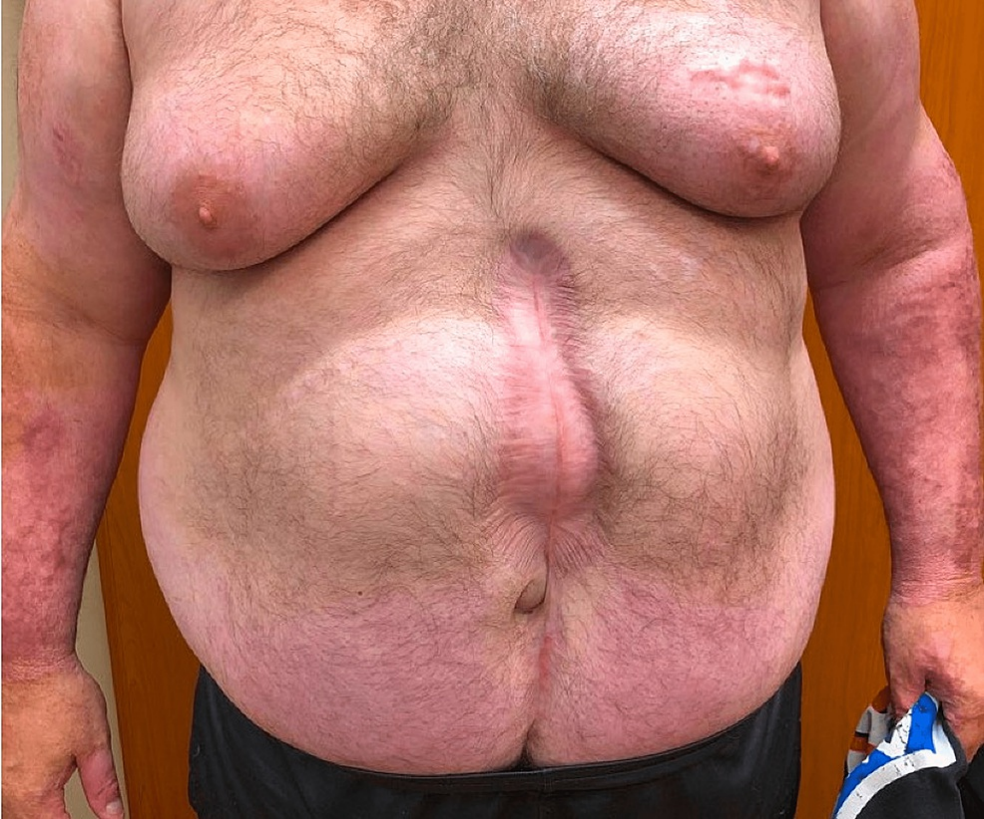
Preoperative physical exam

**Figure 2:**
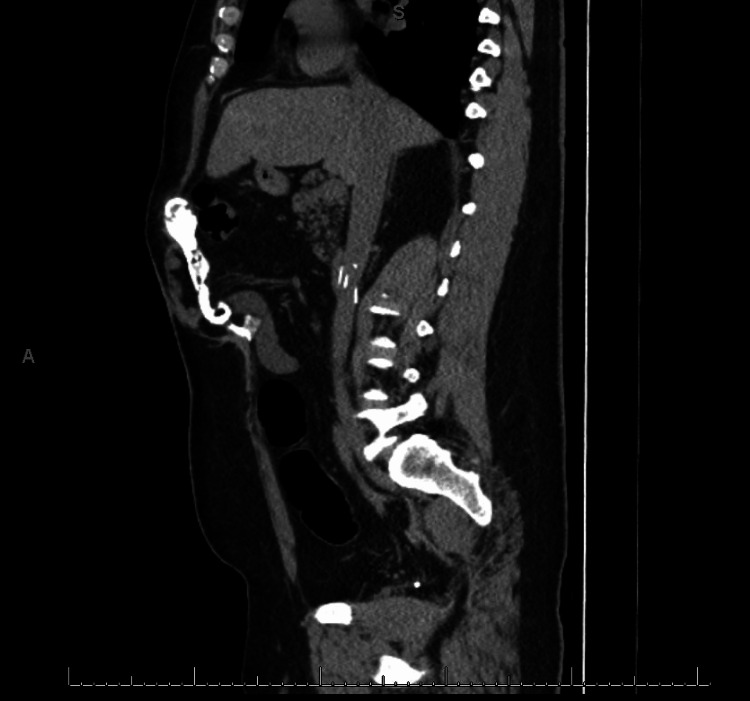
Computer tomography lateral view of the calcified mass

The patient was a 42-year-old man with a history of 55% total burn surface area (TBSA) second and third-degree flame burns. His hospitalization course was complicated by acute colonic pseudo-obstruction with cecal perforation, which required exploratory laparotomy and right hemicolectomy. A temporary abdominal closure device was placed, and he was subsequently closed. During this hospitalization, the postoperative course was complicated by bilateral lower extremity deep venous thrombosis with a saddle pulmonary embolism, which was treated with an inferior vena cava filter and apixaban. Two years after the accident, he presented to our outpatient clinic for the evaluation of an incisional hernia. His physical exam was significant for a body mass index (BMI) of 45 and a healed midline scar with a large mid-abdominal ventral incisional hernia (Figure 1). Computer tomography (CT) imaging demonstrated a large upper midline ventral hernia defect with herniation of the small bowel and omental fat. The hernia width was 10 cm. Additionally, a dense 14.7 cm height x 5.2 cm length x 2.5 cm depth calcification that extended from the xiphoid process along the linea alba and beneath the rectus sheath was noted (Figure 2). After reaching a BMI of less than 40 with dietary and lifestyle modification, he was scheduled for open abdominal wall reconstruction with possible transversus abdominis release and calcified soft tissue mass excision. Apixaban was held preoperatively for three days. 

**Figure 3:**
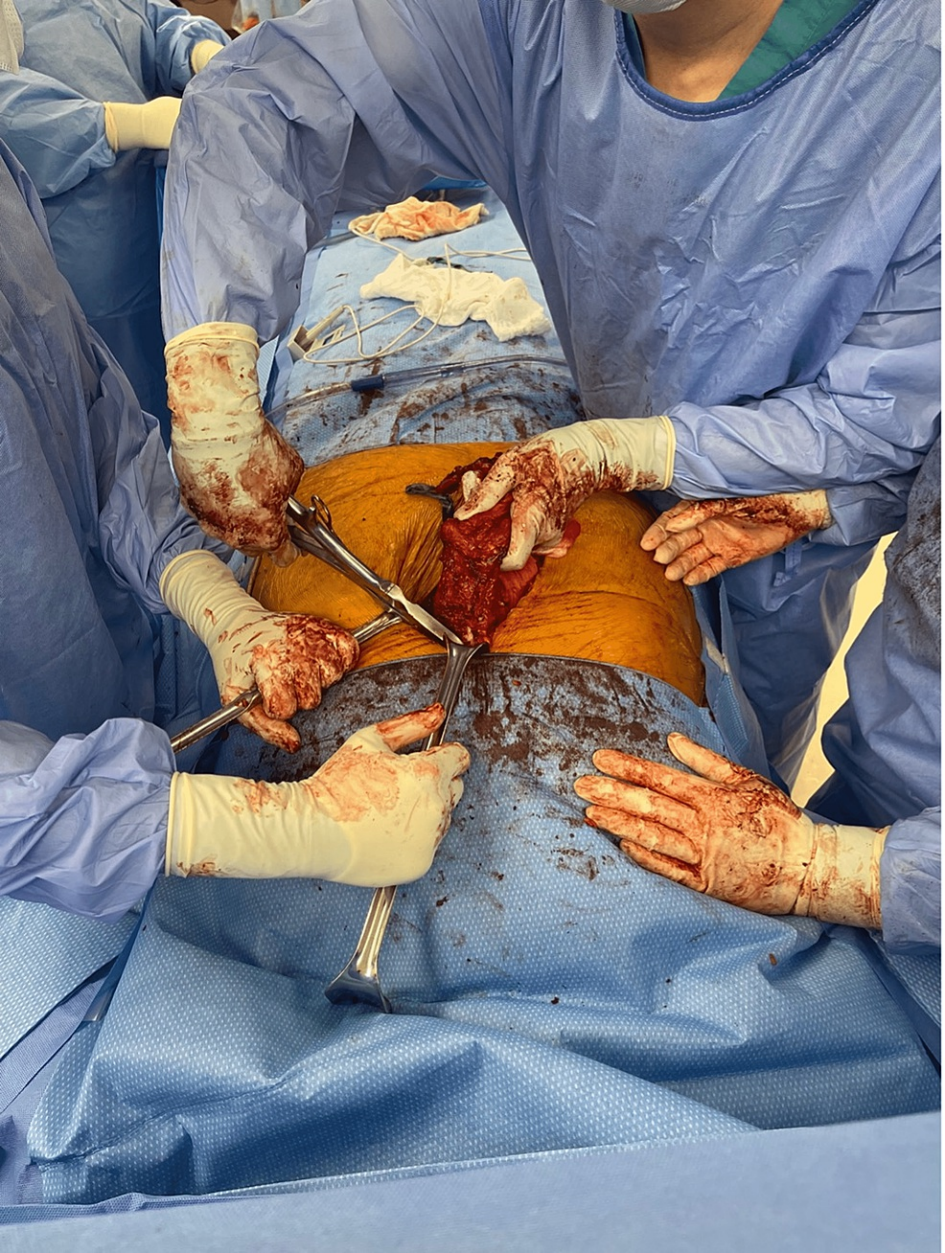
Intraoperative removal of calcified mass

**Figure 4:**
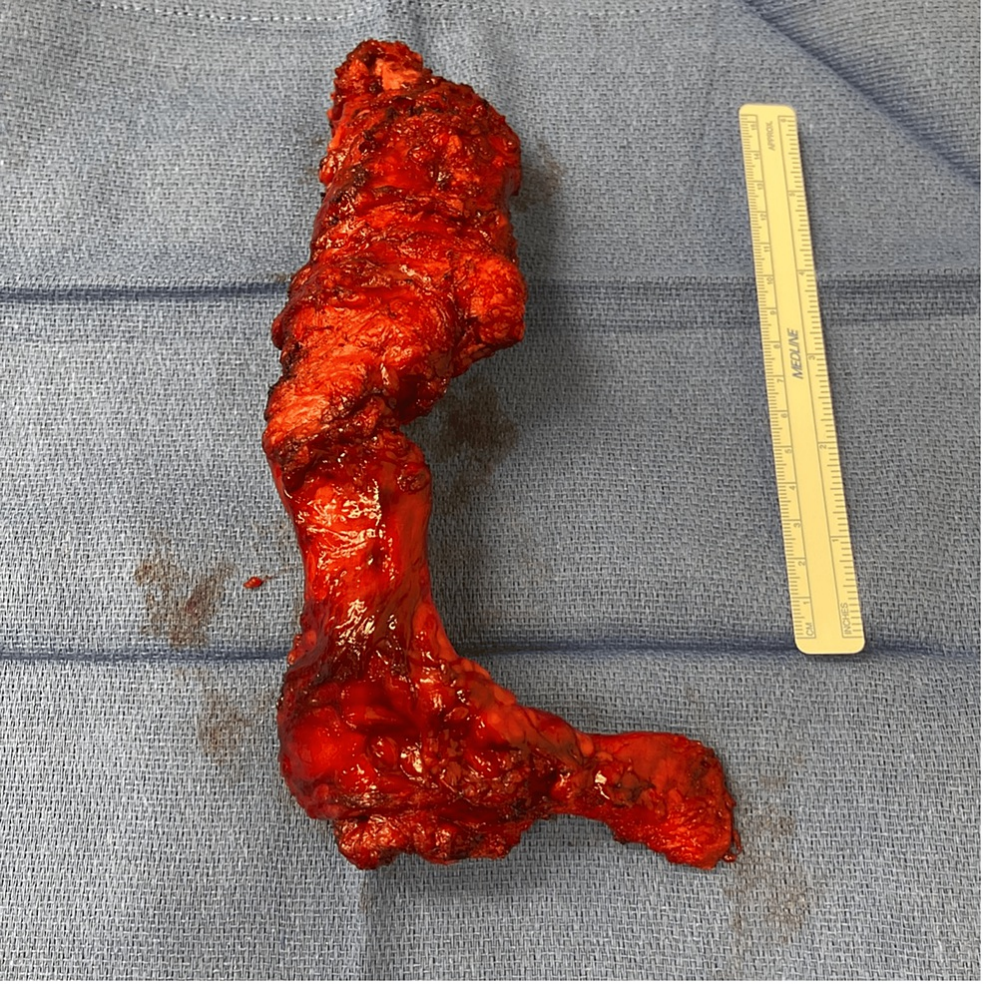
Intraoperative findings

**Figure 5:**
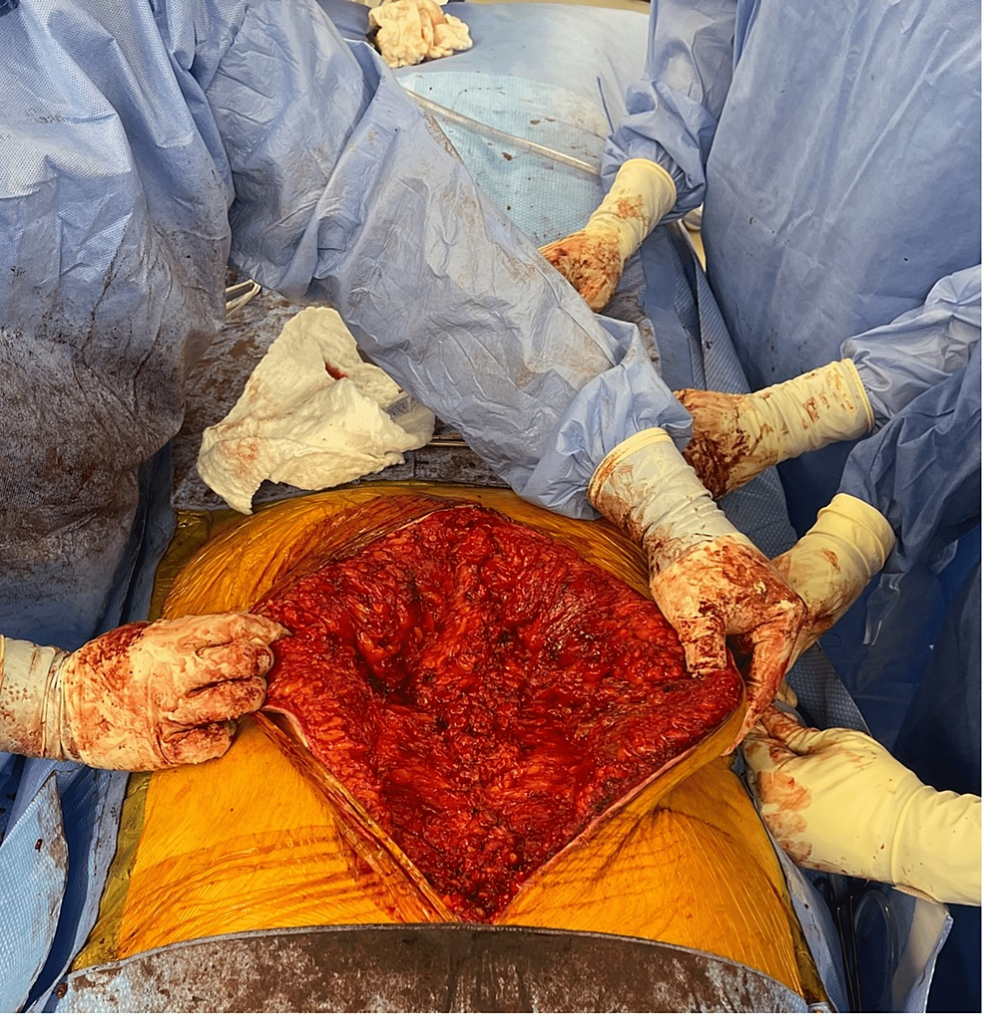
Abdominal wall defect after foreign body extraction

The abdomen was accessed through a midline incision made over the previous scar and carried down to the peritoneum. During this time, a solid calcified soft tissue mass was noted in the soft tissue. The mass was completely dissected up to the xiphoid and then transected from the bone (Figures 3-5). The specimen was sent to pathology. Attention was turned to the abdominal cavity. Dense adhesions to the anterior abdominal wall were released and the right retrorectus space was accessed through a medial incision over the right posterior rectus sheath. The retrorectus space was developed to the linea semilunaris. A top-down transversus abdominis release (TAR) was initiated by incising the posterior lamella of the internal oblique aponeurosis medial to the linea semilunaris and dividing the underlying transversus abdominis muscle fibers. The pretransversalis space was developed and carried downwards to the space of Bogros. The subxiphoid and subcostal retromuscular spaces were also developed. The left retromuscular space was developed in a similar manner. The posterior layer of the rectus sheath was reconstructed using 2-0 Polydioxanone (PDS) in a running fashion. The final hernia defect size measured 10 cm wide and 25 cm long. A medium-weight macroporous polypropylene mesh was selected for reinforcement of the repair. The retromuscular space measured 40 cm x 35 cm. The mesh was trimmed to size and placed in the retromuscular space. Two drains were placed in the retromuscular space. The linea alba was reconstructed using an interrupted figure of 8 sutures with 0-PDS. A third 19 french Blake drain was placed in the subcutaneous space. Postoperatively, the patient had a slow return of bowel function and tolerance to oral intake and was discharged on postoperative day 7. All drains were removed prior to discharge.

**Figure 6:**
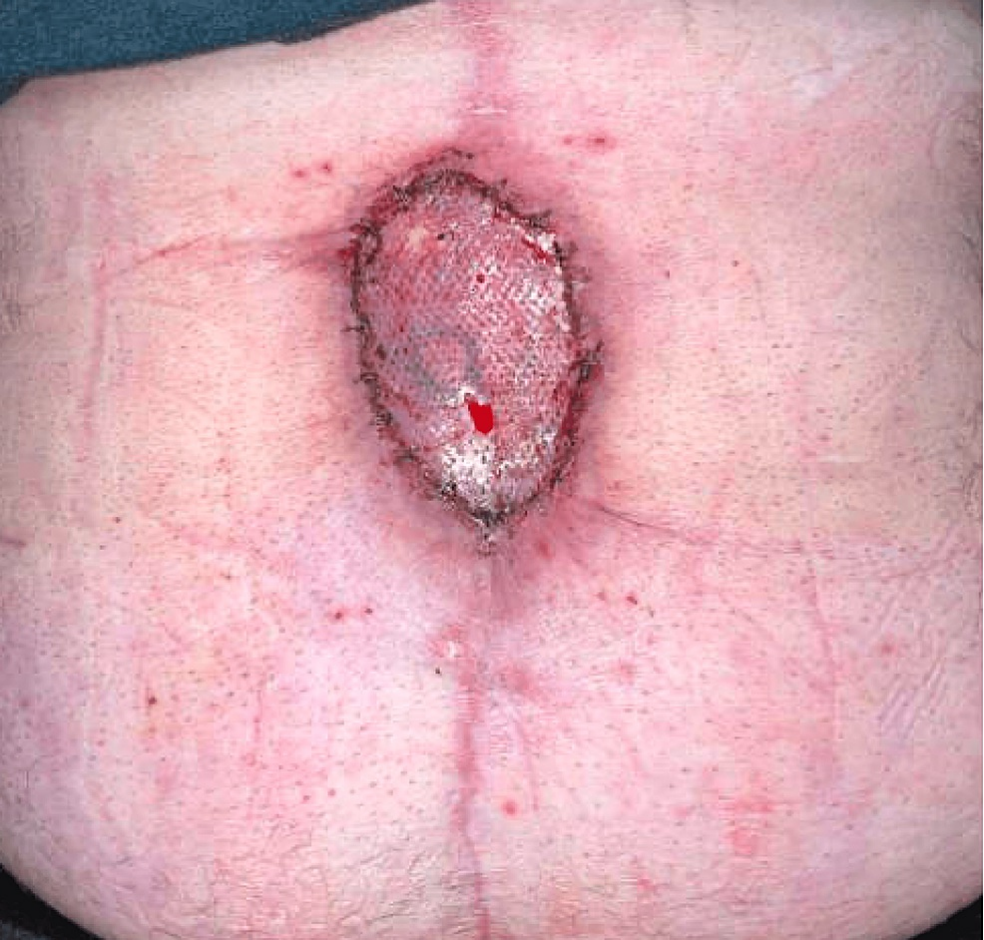
Abdominal wall surgical incision after the skin graft

**Figure 7:**
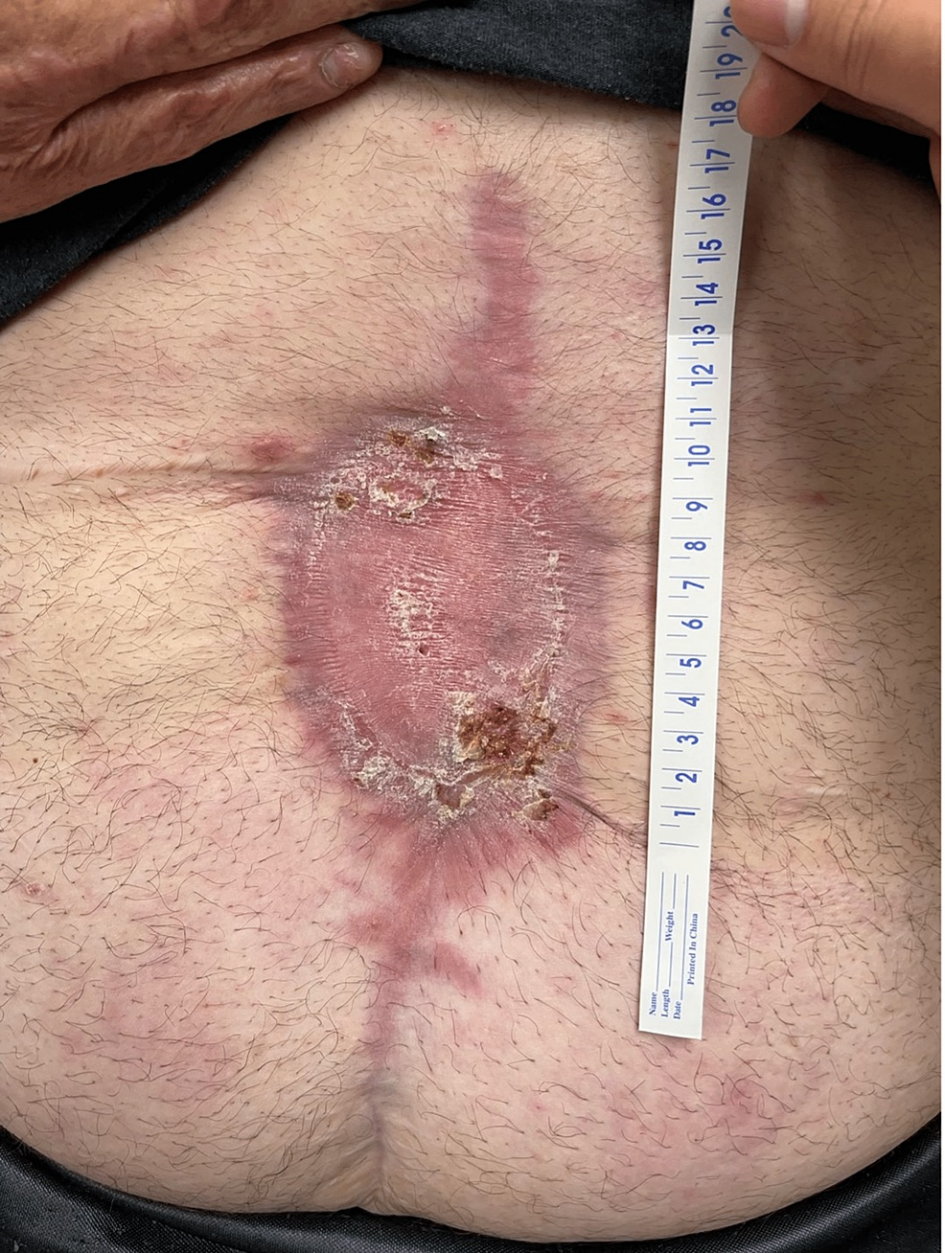
Follow-up visit

Pathology analysis of the mass was consistent with the ossification of soft tissue. The postoperative course was complicated by an infected abdominal wall hematoma anterior to mesh noted on postoperative day 11 after the patient presented with abdominal pain. Further laboratory workup and images conducted were concerning for infection given leukocytosis of 12.6 x10^3^. The patient was taken to the operating room. During the wound wash-out, it was noted that the anterior fascia had partially dehisced exposing the mesh. The mesh was noted to have adequate posterior incorporation and about 20% of the total anterior surface exposed. Negative pressure wound therapy was applied. The patient was discharged home after tolerating oral intake. He then continued to follow up as an outpatient. His wound progressed well and eventually underwent a split-thickness skin graft from the thigh (Figure 6). Five weeks after the graft procedure, he was seen in the clinic. The graft had incorporated without issues and the abdominal wall was completely closed (Figure 7). Five months after his graft procedure, the patient was doing well without further complications or clinical signs of hernia recurrence.

## Discussion

Although the pathophysiology of HO is still unclear, a series of events that create a microenvironment prone to ossification have been described [1,2]. The increase in vasodilation, growth factor accumulation, and cytokine deployment associated with soft tissue hypoxia result in the migration of pluripotent cells and osteogenic precursors [1,2,6]. As a response to injury, these cells express properties that result in osteogenesis [1,2,7]. The aim to preserve and rebuild injured tissue, especially after severe homeostatic disturbances, such as those caused by burn injuries, creates the appropriate setting to develop HO [1]. As explained, midline abdominal incisions associated with HO result from the osseous cell precursors accumulated in areas of major inflammation [8].

Unfortunately, after suffering a burn injury the options to prevent and treat HO are quite limited [7,9]. General preventive strategies include prophylactic non-steroidal anti-inflammatory drugs (NSAIDs) and radiotherapy, however, the safety and efficacy of these tactics are still unclear [7]. For the majority of symptomatic cases, surgical excision is the mainstay of treatment [1,7].

While HO rarely develops in the abdomen, it often involves the xiphoid, especially with a prior midline incision adjacent to the bone [9,10]. This is supported by the handful of literature reporting abdominal ossification where xiphoid involvement is seen [9,10]. Even more infrequent is the presence of HO within an abdominal wall defect. Suleiman et al. described the case of a male with a 13 cm ossified lesion after a midline laparotomy procedure three years prior [10]. This patient underwent ventral hernia repair with Strattice mesh anterior component separation [10]. Although no recurrence was noted at the six-month follow-up, the use of biologic mesh in ventral hernia repair has been associated with higher recurrence rates and up to a 200x increase in cost with no difference in postoperative complications when compared to synthetic mesh [11,12].

Similarly, Akinbiyi et al. reported the case of a 69-year-old male with HO involving the rectus fascia and bowel requiring ventral hernia repair with bilateral anterior component separation and local advancement flap of the anterior rectus sheath [5]. In this situation, the use of mesh was forgone likely due to the presence of multiple enterotomies. The use of mesh in patients undergoing ventral hernia repair has been associated with significantly less recurrence up to 10 years postoperatively when compared to primary repair [13]. Even in the setting of contamination, synthetic mesh use has been described in the single-stage repair of contaminated ventral hernias and could potentially have been used to decrease recurrence in the long term [12]. Despite this, no clinical evidence of recurrence was noted at the 18-month follow-up.

To the best of our knowledge, our case is the first to describe the use of synthetic mesh and transversus abdominis release in a patient with a ventral incisional hernia associated with HO. This component separation technique was first described by Novitsky et al. and has been shown to provide greater anterior myofascial advancement when compared to anterior component separation [14,15]. In addition, it decreases the need for lipocutaneous flap mobilization, as the mesh is placed in a retromuscular position. Overall, posterior component separation has been associated with a lower incidence of surgical site occurrence and surgical site infection when compared to anterior component separation [14,16].

We believe that our patient was at high risk for bleeding and infectious complications given his BMI and use of apixaban. He was severely symptomatic and was optimized prior to surgical intervention. Still, he developed an infected subcutaneous hematoma requiring surgical washout and negative pressure wound therapy. Despite this complication, the patient's symptoms significantly improved and there were no signs of recurrence at nearly six months. Since there are no established guidelines for patients with HO, learning about alternate strategies will expand the armamentarium of abdominal wall reconstruction surgeons in this challenging patient population.

## Conclusions

This case report demonstrates that retromuscular ventral hernia repair with transversus abdominis release and synthetic mesh can be used in complex ventral hernia repair complicated by heterotopic ossification. Physicians performing abdominal wall repairs with HO should be familiar with this option to avoid hernia recurrence and ensure ectopic bone excision.

## References

[1] Ranganathan K, Loder S, Agarwal S (2015). Heterotopic ossification: basic-science principles and clinical correlates. J Bone Joint Surg Am.

[2] Hu X, Sun Z, Li F, Jiang C, Yan W, Sun Y (2021). Burn-induced heterotopic ossification from incidence to therapy: key signaling pathways underlying ectopic bone formation. Cell Mol Biol Lett.

[3] Shehab D, Elgazzar AH, Collier BD (2002). Heterotopic ossification. J Nucl Med.

[4] Moon YJ, Jeong SY, Lee KB (2018). Extra-articular soft-tissue calcification after burn injury: a case study. J Burn Care Res.

[5] Akinbiyi T, Kaul S (2017). Heterotopic ossification encountered during a complex ventral hernia repair: case report and literature review. Eplasty.

[6] Sorkin M, Huber AK, Hwang C (2020). Regulation of heterotopic ossification by monocytes in a mouse model of aberrant wound healing. Nat Commun.

[7] Juarez JK, Wenke JC, Rivera JC (2018). Treatments and preventative measures for trauma‐induced heterotopic ossification: a review. Clin Transl Sci.

[8] Sasaki M, Hotokezaka Y, Ideguchi R, Uetani M, Fujita S (2021). Traumatic myositis ossificans: multifocal lesions suggesting malignancy on FDG-PET/CT—a case report. Skeletal Radiol.

[9] Fennema EM, de Boer J, Mastboom WJ (2014). Ossification of abdominal scar tissue: a case series with a translational review on its development. Hernia.

[10] Suleiman NN, Sandberg LJ (2016). Extensive abdominal wall incisional heterotopic ossification reconstructed with component separation and Strattice inlay. Plast Reconstr Surg Glob Open.

[11] Harris HW, Primus F, Young C (2021). Preventing recurrence in clean and contaminated hernias using biologic versus synthetic mesh in ventral hernia repair: the PRICE randomized clinical trial. Ann Surg.

[12] Rosen MJ, Krpata DM, Petro CC (2022). Biologic vs synthetic mesh for single-stage repair of contaminated ventral hernias: a randomized clinical trial. JAMA Surg.

[13] Burger JW, Luijendijk RW, Hop WC, Halm JA, Verdaasdonk EG, Jeekel J (2004). Long-term follow-up of a randomized controlled trial of suture versus mesh repair of incisional hernia. Ann Surg.

[14] Novitsky YW, Elliott HL, Orenstein SB, Rosen MJ (2012). Transversus abdominis muscle release: a novel approach to posterior component separation during complex abdominal wall reconstruction. Am J Surg.

[15] Majumder A, Martin-Del-Campo LA, Miller HJ, Podolsky D, Soltanian H, Novitsky YW (2020). Evaluation of anterior versus posterior component separation for hernia repair in a cadaveric model. Surg Endosc.

[16] Maloney SR, Schlosser KA, Prasad T (2019). Twelve years of component separation technique in abdominal wall reconstruction. Surgery.

